# Effect of Post-Printing Methods on the Microstructure and Mechanical Properties of Ti6Al4V Titanium Alloy Samples Fabricated Using Laser Powder Bed Fusion

**DOI:** 10.3390/ma19020401

**Published:** 2026-01-19

**Authors:** Krzysztof Żaba, Stanislav Rusz, Alicja Haslik-Sopata, Łukasz Kuczek, Ilona Różycka, Maciej Balcerzak, Tomasz Trzepieciński

**Affiliations:** 1 Department of Metal Working and Physical Metallurgy of Non-Ferrous Metals, Faculty of Non-Ferrous Metals, AGH University of Krakow, al. Adama Mickiewicza 30, 30-059 Cracow, Poland; ahaslik@interia.pl (A.H.-S.); lukasz.kuczek@agh.edu.pl (Ł.K.); balcerzak@agh.edu.pl (M.B.); 2 Faculty of Mechanical Engineering, VSB-Technical University of Ostrava, 17. listopadu 2172/15, 708 00 Ostrava-Poruba, Czech Republic; stanislav.rusz@vsb.cz; 3 Department of Materials Science and Engineering of Non-Ferrous Metals, Faculty of Non-Ferrous Metals, AGH University of Krakow, al. Adama Mickiewicza 30, 30-059 Cracow, Poland; rozycka@agh.edu.pl; 4 Department of Manufacturing Processes and Production Engineering, Faculty of Mechanical Engineering and Aeronautics, Rzeszów University of Technology, al. Powst. Warszawy 8, 35-029 Rzeszów, Poland; tomtrz@prz.edu.pl

**Keywords:** LPBF, HIP, TCAP, microhardness, mechanical properties, microstructure, Ti6Al4V

## Abstract

Laser powder bead fusion (LPBF) allows for the fabrication of highly accurate components from metal powders, which is difficult to achieve using traditional methods. LPBF-produced components can be characterized by their porosity and unfavorable microstructure, making further processing difficult. Therefore, appropriate post-printing methods are crucial, as they reduce porosity, reduce residual stresses, and stabilize the microstructure. The aim of this paper was to determine the effect of post-printing methods on the microhardness and microstructure of Ti6Al4V titanium alloy samples fabricated using the LPBF process in different orientations. Hot isostatic pressing (HIP) at various temperatures (910 °C, 1150 °C, 1250 °C), annealing at 1020 °C, and twist channel angular pressing using a 90° channel ending with a helical exit were considered postprocessing methods for LPBF-produced samples. Printing orientation significantly determined the effectiveness of HIP and the heat treatment processes. Higher microhardness was observed on the cross-section oriented perpendicular to the 3D printing direction. Annealing under appropriately selected conditions favors the precipitation of fine particles of the α phase in the β phase, leading to a strengthening effect by precipitation. Based on the microhardness measurements, clear differences were observed in the mean values, statistical ranges, and result distributions depending on the printing plane, HIP process parameters, and the use of an additional heat treatment. The HIP process leads to a more pronounced homogenization of microstructure and defect reduction, with the morphology of the microstructure and microhardness distribution dependent on the HIP process temperature.

## 1. Introduction

The Ti6Al4V titanium alloy is the most widely used titanium material in engineering applications requiring critical requirements for strength, mass, and corrosion resistance [1]. Its popularity stems from its two-phase α + β microstructure and the deliberate selection of phase stabilizers: aluminum (α stabilizer) and vanadium (β stabilizer) [2]. Ti6Al4V is difficult to machine using conventional methods. Alternative manufacturing methods of difficult-to-deform materials are increasingly being sought, which reduce material loss, shorten the process chain, and enable component personalization [3]. In this context, additive manufacturing (AM) techniques become a natural choice, as they build components layer-by-layer without the need for machining [4]. This enables the fabrication of prototypes and tools (rapid tooling), as well as finished products (rapid manufacturing). The most popular AM methods for manufacturing metal components include selective laser melting (SLM) [5], direct metal laser sintering (DMLS) [6], and electron beam melting (EBM) [7], where layers of metal powder are melted using a laser or electron beam. The advantage of AM techniques is the ability to form internal channels in scaffold structures that are practically unattainable using conventional processes (casting, plastic working, machining, etc.) [8]. Laser Powder Bed Fusion (LPBF) is a process for the selective melting of thin powder layers using a laser beam in an inert gas shield [9]. A growing number of studies have indicated that properly selected LPBF processing and postprocessing methods can enable the production of the components characterized by mechanical properties which are comparable to, and sometimes even better than, cast components [10,11]. At the same time, very high heating/cooling rates and steep thermal gradients in LPBF generate microstructures that deviate from equilibrium states.

LPBF primarily utilizes metallic materials such as stainless steel, titanium, aluminum, cobalt–chromium alloys, and nickel alloys, as well as some ceramics and polymers. The titanium alloy Ti6Al4V, with its combination of excellent strength-to-weight ratio, biocompatibility, and corrosion resistance, is the most commonly used material in LPBF processes [12]. The most characteristic feature of LPBF-processed Ti6Al4V material is the tendency to form a martensitic α′ phase due to rapid cooling from the β region and the formation of elongated prior-β grains [13]. The formation of the α′ phase is accompanied by high residual stresses and material anisotropy resulting from directional grain growth [2]. Additionally, LPBF process defects may remain in the structure of the workpiece material—micropores, lack of fusion, and interlayer discontinuities—which is particularly unfavorable for fatigue life [14]. The fatigue life of AM-produced Ti6Al4V alloy depends on crack initiation from microstructural features [15]. Furthermore, the hard α′ phase increases the material’s strength while reducing ductility [16]. This poses a problem for the subsequent processing of LPBF-produced components. Therefore, appropriate post-printing methods are crucial, as they reduce porosity and stabilize the microstructure [17].

The first post-printing process considered in this paper is hot isostatic pressing (HIP). This process elevates the temperature and isostatic inert gas pressure, diffusion sealing the internal porosity and homogenizing the microstructure, to improve the density, uniformity, and fatigue properties of the material. However, it is important to note that HIP effectiveness depends on the print quality, the LPBF-produced component, and HIP process parameters. Standard HIP treatments of Ti6Al4V fall below the β-transus [18], which results in a decrease in tensile strength (from a Hall–Petch effect [19]). Excessively high temperatures can lead to thickening of the α phase and a reduction in material strength, while properly selected parameters allow for a significant improvement in material durability. It has been demonstrated that the morphology of the α and β phases in the Ti6Al4V alloy undergoes spheroidization after HIP, with a simultaneous increase in grain size [20].

The second stage of post-printing processing analyzed in this paper is additional heat treatment (annealing) after HIP. Its purpose is to control phase transformations in order to transform the acicular α martensite into an α + β microstructure. Heat treatment (HT) in the post-printing phase is particularly important for optimizing the mechanical properties of LPBF-produced components [21]. Annealing also allows for the dissolution or rearrangement of α’ needles and the thickening of α plates and stress relaxation, which increases ductility without excessively reducing the strength of Ti6Al4V alloy. Properly designed thermal cycles improve ductility and toughness while maintaining high strength, providing a better overall balance of mechanical performance [22]. Common heat treatments include solution treatment and aging to form a stable α + β microstructure and annealing treatments below the β-transus to promote fine lath microstructures and improve elongation.

The third post-printing process considered in this work is twist channel angular pressing (TCAP) [23], which is a severe plastic deformation (SPD) method that allows for the introduction of very large shear strains without changing the sample cross-section [24]. TCAP leads to strong grain refinement and the formation of a dense dislocation network, resulting from work hardening [25] and, with appropriate parameter control, an improvement in the strength–ductility balance [26]. In the case of Ti6Al4V, this process can improve the mechanical properties of materials, provided that the material has adequate deformability provided by prior HIP and annealing operations. The sequence of processes—from 3D printing using the LPBF method, through HIP and additional annealing, to TCAP—creates a coherent system for controlling the microstructure and properties of the Ti6Al4V titanium alloy. The 3D printing process defines the initial state of the material, HIP reduces defects and homogenizes the structure, heat treatment stabilizes the phases and increases plasticity, and TCAP allows for the further improvement in mechanical properties. This approach not only reduces porosity but also achieves a favorable compromise between hardness and ductility, which directly translates into component reliability in demanding applications. In light of the above, the aim of this study is to thoroughly assess the impact of post-printing methods (including HIP, additional heat treatment, and TCAP) on the microstructure, microhardness, and porosity of LPBF-produced Ti6Al4V alloy bars.

## 2. Materials and Methods

### 2.1. Material

Spherical Ti6Al4V powder, supplied by Linde Advanced Material Technologies Inc. (Danbury, CT, USA), was used to produce the samples using the LPBF method. The morphology of the metal powder was determined using SU-70 scanning electron microscopy (SEM) (Hitachi, Tokyo, Japan). Analysis of the Ti6Al4V particle morphology revealed that their sizes ranged between 30 and 50 μm. The chemical composition of Truform powder was determined by the manufacturer Praxair Surface Technologies (Speedway, IN, USA). Titanium forms a matrix that provides strength and corrosion resistance. Aluminum strengthens and stabilizes the α phase and reduces the alloy density, thereby increasing corrosion resistance and stiffness. In turn, vanadium acts as a β phase stabilizer, simultaneously increasing the material’s ductility.

### 2.2. Laser Powder Bed Fusion

A RenAM 500S Flex machine (Renishaw plc, Woodchester, UK) was used to produce 15 × 15 × 60 mm rectangular samples using the LPBF method. The processing parameters presented in Table 1 were selected based on the RenAM 500S Flex machine documentation guidelines and preliminary tests.

**Figure 1:**
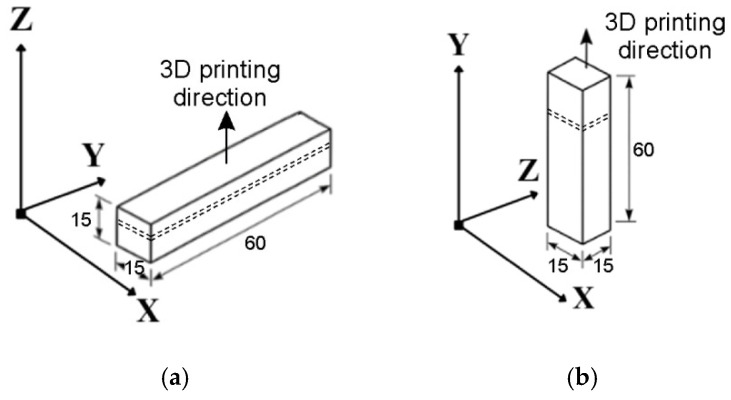
LPBF printing scheme in planes: (**a**) XY and (**b**) XZ.

The use of additive manufacturing technologies enables precise manufacturing of components, but their final properties are strongly dependent on the 3D printing direction. Our samples were printed in the XY plane (Figure 1a) and the XZ plane (Figure 1b).

The properties of the LPBF-produced samples were assessed in the as-printed condition, after HIP at temperatures ranging from 910 to 1250 °C, and after additional heat treatment (HT). In addition, selected samples were subjected to the TCAP method.

### 2.3. Hot Isostatic Pressing

HIP was performed at various temperatures (910 °C, 1150 °C, 1250 °C) for 240 min. For titanium alloys, high-vacuum processing is recommended, preferably without shielding gases. Tests were conducted using a vacuum pressure of 200 MPa, and the research plan with individual checkmarks is presented in Table 2.

### 2.4. Heat Treatment

**Figure 2:**
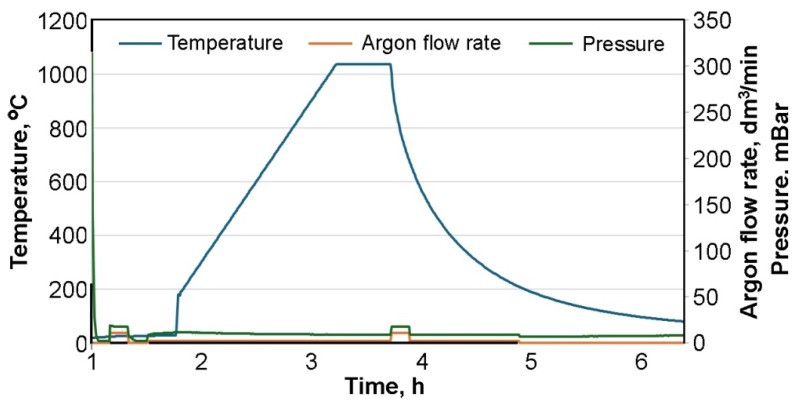
Heat treatment parameters of LPBF-processed Ti6Al4V samples.

LPBF-produced Ti6Al4V components exhibit significantly higher tensile strength and yield strength values than the ASTM specification; therefore, heat treatment is typically performed on 3D-printed Ti6Al4V components [27]. As part of the experimental study, heat treatment of HIP-processed samples was performed. The purpose of the heat treatment (annealing) was to reduce the work hardening effects induced by HIP. It should be noted that the sample surfaces were already oxidized before the heat treatment process. Heat treatment was performed at 1020 °C, at 10 °C/min and held at this temperature for 1 h, and then slowly cooled to room temperature (RT) inside the furnace (Figure 2) under non-vacuum conditions and in an argon atmosphere. To obtain comparable conditions in both the XY and XZ 3D printing planes, samples 3XY, 4XY, 7XZ, and 8XZ were also heat-treated under the same conditions and designated as 3XY-HT, 4XY-HT, 7XZ-HT, and 8XZ-HT (Table 3).

### 2.5. Twist Channel Angular Pressing

**Figure 3:**
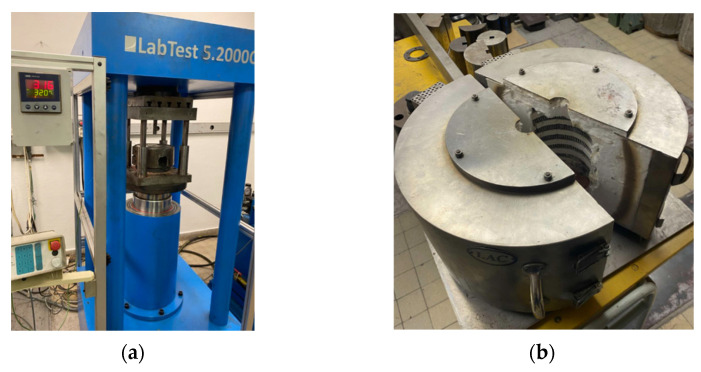
(**a**) LabTest 5.2000CT press and (**b**) sample heating chamber.

To obtain an ultrafine-grained microstructure that would allow for a balance between the strength and plastic properties of the LPBF-processed and HIP-processed samples, the samples were deformed using the TCAP method. The tests were conducted at the Department of Mechanical Technology at the VSB-Technical University of Ostrava. The deformation of samples measuring 15 mm × 15 mm × 60 mm was performed using a LabTest 5.2000CT hydraulic press (LabControl, Opava, Czechia) through the TCAP die (Figure 3a) with a maximum force of 2000 kN. The samples were heated to the required temperature (350 °C) using a heating chamber (Figure 3b).

**Figure 4:**
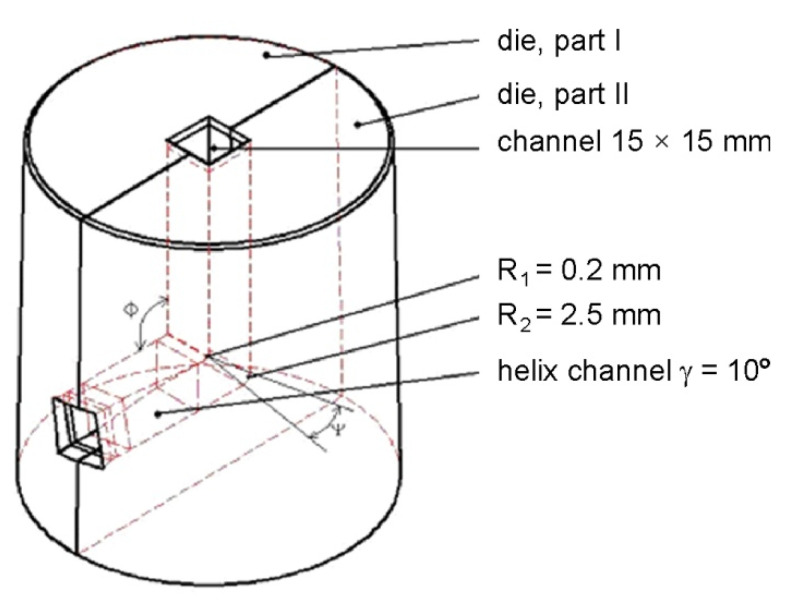
Schematic of the TCAP tool.

**Figure 5:**
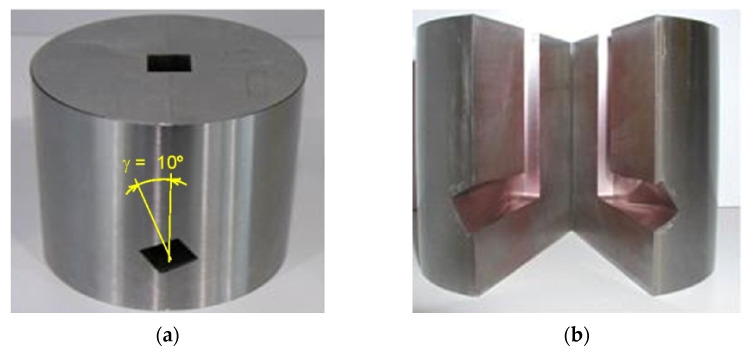
(**a**) Side view of the TCAP die and (**b**) cross-section of the TCAP die with a helix channel.

From the point of view of ensuring a uniform strain distribution, the most effective solution of the TCAP tool is a 90° channel ending with a helical exit (Figure 4) [28]. The basic channel geometry is defined by the angles Φ = 90° (internal channel angle) and Ψ = 9.5° (external channel angle). Angle Ψ results from the selected radii with values R_1_ = 0.2 mm and R_2_ = 2.5 mm. The helical channel inclination angle used was γ = 10° (Figure 5).

The TCAP die was made of HOTVAR^®^ tool steel (Böhler-Uddeholm, Vienna, Austria). The tensile strength of HOTVAR^®^ steel is at least 2100 MPa, and the hardness is at least 54 HRC.

The 2XY sample was subjected to HIP at 910 °C (Table 2), and the 5XZ sample in the as-printed state was heated to 350 °C. During the process, the forming tool also had to be heated to the desired forming temperature. For this purpose, an M20VA induction furnace (LAC s.r.o., Židlochovice, Czechia) was used, with a maximum heating temperature of 1150 °C. The heating device was controlled by a dTRON 304 device (JUMO GmbH & Co. KG, Fulda, Germany) with a NiCr-Ni thermocouple, which can be used up to a maximum temperature of 1350 °C.

To minimize the influence of friction on the inhomogeneity of the TCAP-processed material, Thermocup 1200 graphite lubricant (Nicro Kft., Vecsés, Hungary) was used. The TCAP speed was 40 mm/min.

### 2.6. Microstructure Examination

Microstructural observations of the samples produced by the LPBF method were performed using a Stemi 305 optical microscope (Zeiss, Oberkochen, Germany) and an S-3400 N SEM-EDS (Hitachi, Tokyo, Japan). The analysis was performed at an accelerating voltage of 20 kV and a working distance of 10 mm, using a Condenser 1 (C1) setting of 55,200, optimized for EDS acquisition. Under these operating conditions, the electron probe spot size for EDS analysis was approximately 100–500 nm. It should be emphasized that despite the sub-micrometer electron probe size, the effective spatial resolution of EDS at 20 kV is governed by the electron–matter interaction volume. Therefore, the recorded EDS signal represents the averaged composition over this interaction volume rather than the nominal beam spot size.

Metallographic samples were ground using abrasive paper with grits ranging from 250 to 4000 µm. The ground samples were then polished on polishing cloths using DiaDuo diamond pastes with grits ranging from 6 to 3 µm (Struers, Copenhagen, Denmark). Then, samples were polished in a colloidal silica-based polishing suspension (OP-S) provided by Struers (Copenhagen, Denmark). The polished samples were etched with Keller’s reagent (4 parts HF, 6 parts HCL, 8 parts HNO_3_, and 82 parts H_2_O).

### 2.7. Microhardness Testing

**Figure 6:**
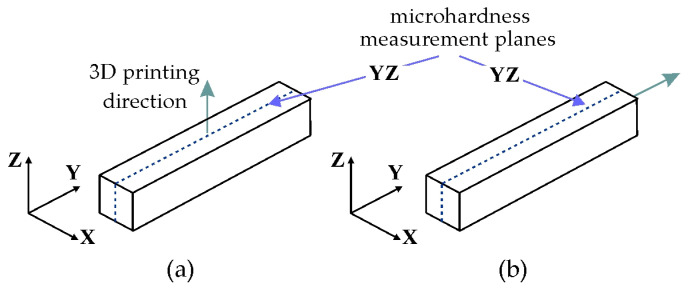
Microhardness measurement planes for samples printed in (**a**) XY and (**b**) XZ planes.

Microhardness testing of the LPBF-processed and post-printing samples was performed using the Vickers method, in accordance with EN ISO 6507-1 [29]. Measurements were performed on a Tukon 2500 hardness tester (Wilson, NY, USA) under a load of 0.5 kg. To represent the spatial distribution of hardness in the samples, indentations were made at regular 1 mm intervals in the measurement plane. Microhardness was measured in the YZ cross-section of LPBF-printed samples in planes XY (Figure 6a) and XZ (Figure 6b).

### 2.8. Computed Tomography

**Figure 7:**
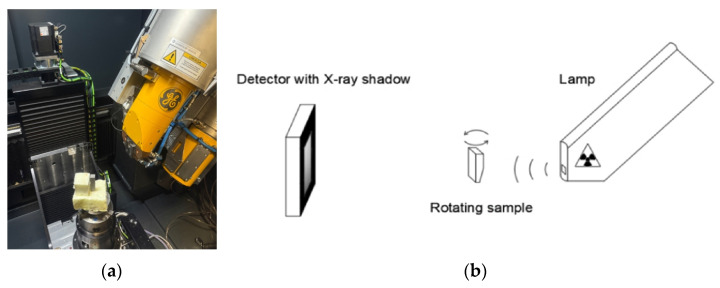
(**a**) Chamber of CT scanner and (**b**) diagram of CT X-ray imaging procedure.

The LPBF-printed samples were subjected to non-destructive testing using X-ray computed tomography with a dual-tube X-ray microtomograph, the GE Phoenix V|tome|X M system (Figure 7a). The inspection process was carried out in a dedicated tomography chamber, in which each specimen was positioned between the radiation source and the detector. The radiation source and the detector remained stationary, while the specimen itself was rotated around the vertical axis (Figure 7b). During each examination, approximately 2000 projections were acquired, and the measurement resolution was 20 µm.

The 3D model reconstruction was performed using correction algorithms such as AGC (Automatic Geometric Calibration) and BHC+ (Beam Hardening Correction) with a filter coefficient of 6.8, enabling automatic calibration and optimization of the system geometry. Further defect analysis was conducted using the VGDefX algorithm, which automatically determined void detection thresholds based on corrected surface histograms, taking into account a reduction factor of −2.25.

## 3. Results and Discussion

### 3.1. Microstructure Analysis

Analysis of the Ti6Al4V particle morphology revealed that their sizes ranged between 30 and 50 μm. The chemical composition is presented in Table 3.

**Figure 8:**
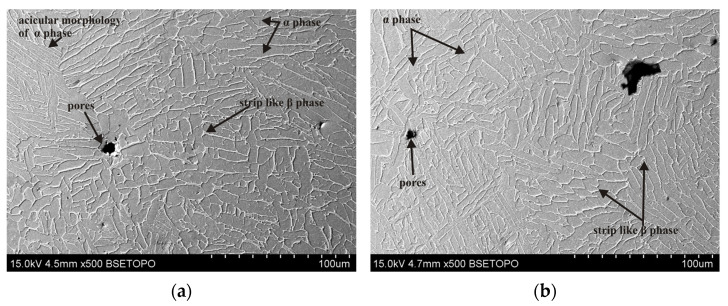
SEM micrograph of the 1XY sample (as-printed condition): (**a**) longitudinal section and (**b**) transverse section relative to the printing direction.

**Figure 9:**
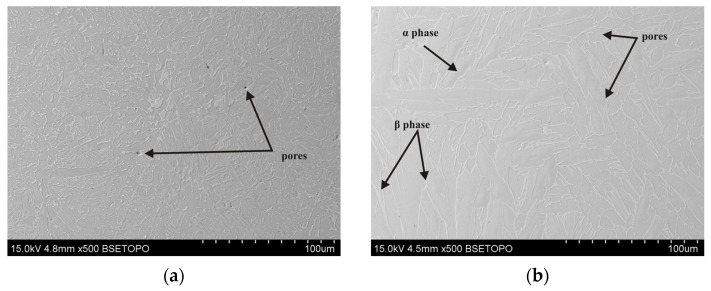
SEM micrograph of the 2XY sample subjected to HIP (910 °C) and TCAP: (**a**) longitudinal section and (**b**) transverse section relative to the printing direction.

**Figure 10:**
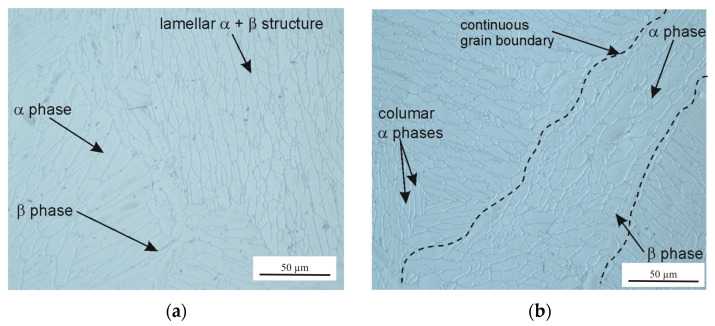
Optical microstructure of the 3XY-HT sample: (**a**) longitudinal section and (**b**) transverse section relative to the printing direction.

**Figure 11:**
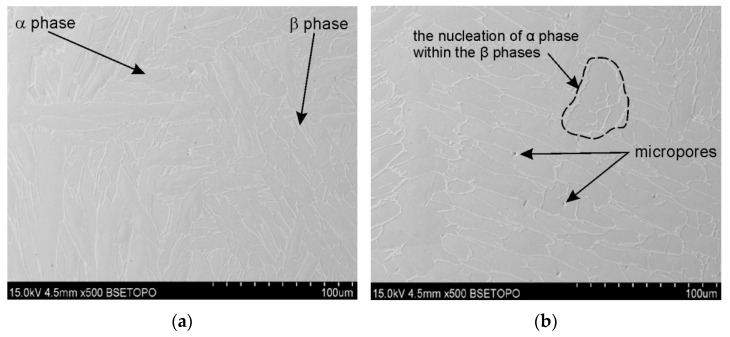
SEM micrographs of the 4XY-HT sample: (**a**) longitudinal section and (**b**) transverse section relative to the printing direction.

The microstructure of the tested Ti6Al4V samples was examined by taking into account different printing plane orientations (XY and XZ) and the post-printing processes: HIP, HT, and TCAP. Due to the repeatability of the obtained microstructural observation results, only selected, representative microstructure SEM micrographs are presented. A strong impact of the 3D printing plane on the microstructure of the LPBF-produced samples was observed. Samples printed in the XY plane show visible porosity bands (Figure 8, Figure 9, Figure 10 and Figure 11) arranged linearly along the laser scanning paths and corresponding to the bonding zones of successive powder layers. The characteristic layered structure leads to the formation of elongated grains and regularly arranged defects, indicating that printing in the XY plane promotes greater microstructural uniformity.

In the as-printed sample (1XY), numerous micropores were observed (Figure 8), which are the result of insufficient melting or the presence of gas trapped in the powder particles. The pores are mainly distributed at the printing layer boundaries. Hot isostatic pressing significantly reduces their number and size, as confirmed by observations of the samples subjected to HIP and heat treatment (Figure 10 and Figure 11). However, even at a pressure of 200 MPa and a HIP temperature of 1250 °C, they cannot be completely eliminated; single, small micropores were observed in the microstructure (Figure 11).

**Figure 12:**
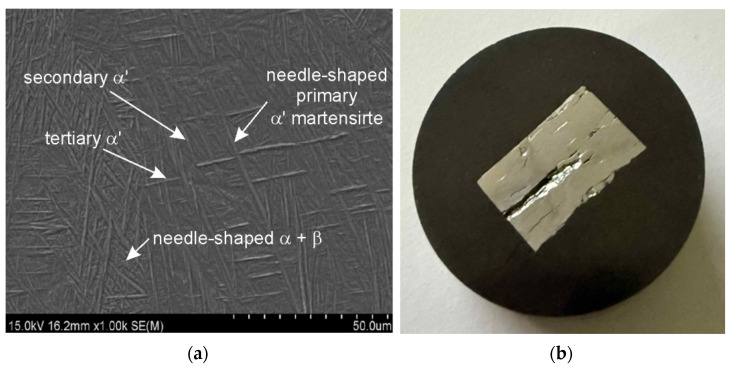
(**a**) SEM micrograph of a transverse section of the 5XZ sample printed in the XZ plane (as-printed state) and (**b**) transverse section view of the 5XZ sample.

**Figure 13:**
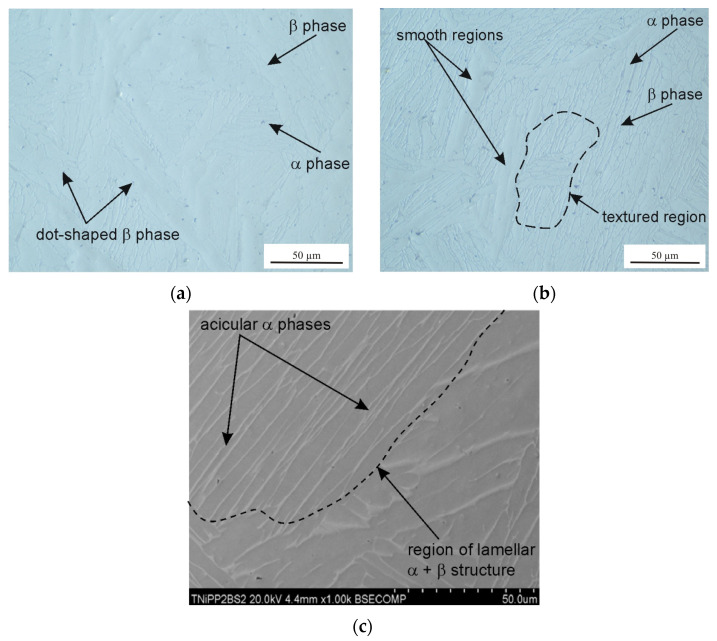
Optical microstructures of the 7XZ-HT sample in (**a**) longitudinal section and (**b**) transverse section relative to the printing direction and (**c**) the SEM micrograph.

**Figure 14:**
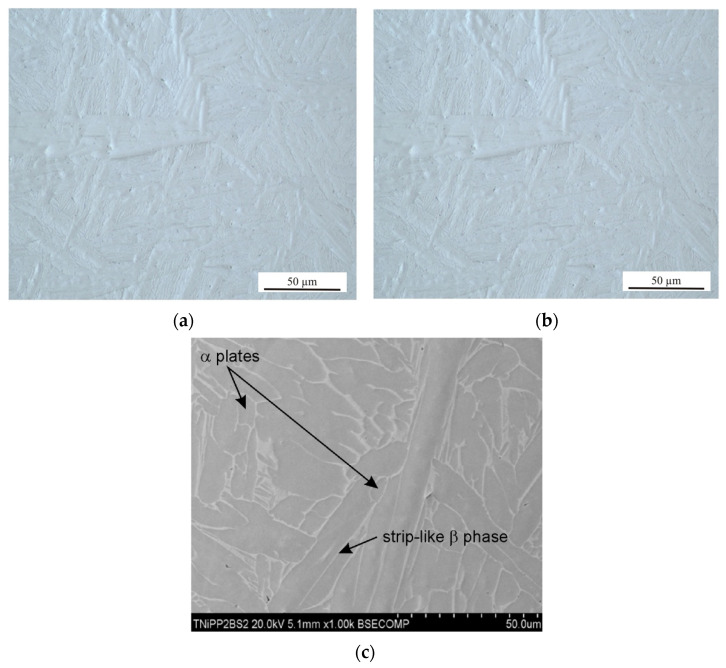
Optical microstructures of the 8XZ-HT sample in (**a**) longitudinal section and (**b**) transverse section relative to the printing direction and (**c**) SEM micrograph.

Samples printed in the XZ plane (Figure 12, Figure 13 and Figure 14) exhibit a different microstructural pattern than the samples printed in the XY plane. Printing in the XY plane results in a more heterogeneous microstructure, with elongated columnar grains and local discontinuities. These differences indicate anisotropy in the sample material, resulting from the printing direction, which impacts both the grain morphology and the character of the defects observed [30,31].

**Figure 15:**
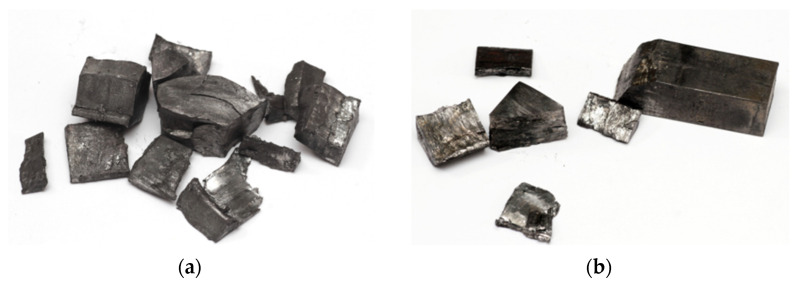
View of the samples after the TCAP process: (**a**) the 2XY sample and (**b**) the 5XZ sample.

The observation of cracks in the samples subjected to the TCAP process is particularly significant. Sample 2XY (Figure 15a) and sample 5XZ (Figure 12b and Figure 15b) suffered extensive damage after this process, which prevented full microstructural analysis. A HIP-processed 2XY sample at 910 °C was damaged during the TCAP process (Figure 15a). To check whether the HIP process was the cause of the cracking, a sample printed in the XZ plane (but without HIP postprocessing) was subjected to the TCAP process. However, the material was damaged in this case as well (Figure 15b). Numerous cracks indicate high stresses generated during severe plastic deformation.

The phase transformation temperature α + β ↔ β in the titanium alloy Ti-6Al-4V is approximately 950 °C, although the β-transus temperature can vary in the range of 700–1050 °C [32]. The precise value of the transformation temperature depends on the chemical composition of the material and the specific processing conditions. Liu et al. [33] determined the β-transus temperature of as-received Ti6Al4V to be 975 ± 5 °C. The HIP process combined with heat treatment and TCAP significantly affects grain morphology. In the 2XY sample subjected to HIP at 910 °C and TCAP (Figure 9), significant grain refinement was observed, which is characteristic of SPD processes. In the 4XY-HT sample, on the other hand, the microstructure became more uniform, and the grain size was reduced, confirming the effectiveness of this combination of post-printing processes (Figure 11). A similar phenomenon was observed for samples printed in the XZ plane. The microstructure of the LPBF-produced Ti6Al4V sample showed the presence of an α′ martensitic phase with a needle-like structure (Figure 12a), which is a consequence of the high cooling rate [27]. In the heat-treated samples above the β transus, the acicular α′ martensite no longer occurs.

The 7XZ-HT and 8XZ-HT samples subjected to HIP and heat treatment (Figure 13 and Figure 14) were characterized by a more homogeneous structure and finer grains than the as-printed samples. In the sample 7XZ-HT subjected to HIP and heat treatment, the β phase rods are discontinuous and a dot-shaped β phase was revealed (Figure 12a), which can be interpreted as the nucleation of the α phase within the β phase [34]. The grain refinement effect improves the homogeneity of the microstructure, which translates into a potential improvement in mechanical properties, especially fatigue strengths [35].

### 3.2. Porosity Analysis

**Figure 16:**
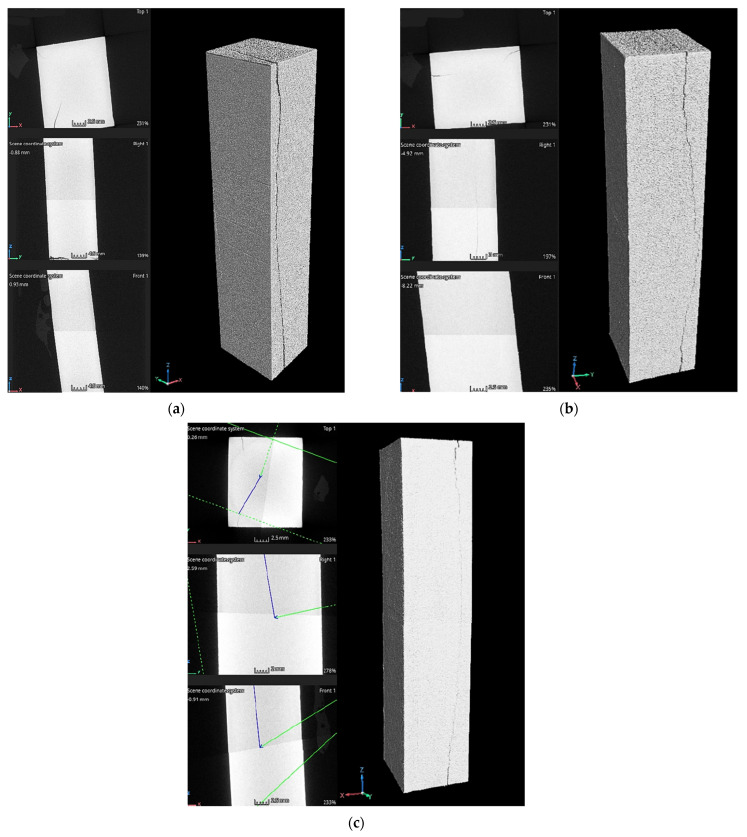
CT scans of the LPBF-printed samples after hot isostatic pressing: (**a**) 2XY, (**b**) 3XY, and (**c**) 4XY.

**Figure 17:**
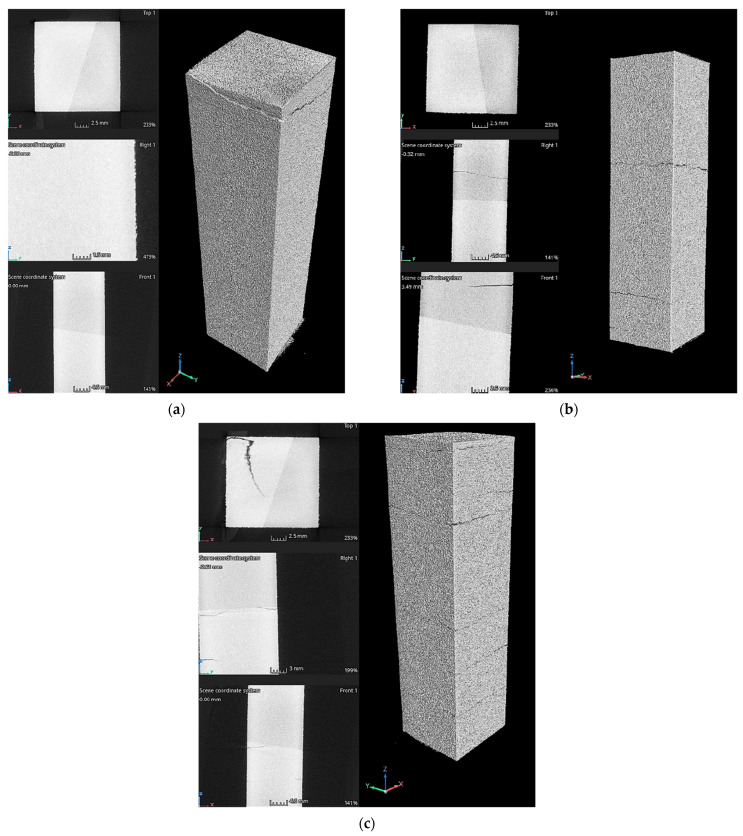
CT scans of the LPBF-printed samples after hot isostatic pressing: (**a**) 6XZ, (**b**) 7XZ, and (**c**) 8XZ.

Based on the obtained tomographic scans, distinct structural differences were observed between the samples depending on their orientation during the printing process. Samples printed in the XY plane (Figure 16) were characterized by a more homogeneous and coherent layer distribution, suggesting a stable process of successive layer formation. The layers were arranged parallel to each other and exhibited no pronounced interlayer boundaries. In contrast, samples printed in the XZ orientation (Figure 17) showed more distinct interlayer boundaries, resulting from cross-sectional observations relative to the direction of successive material deposition. In these cross-sections, local discontinuities were observed, which may be attributed to insufficient melting of the material at the interlayer interfaces. No pores were detected in the material at a scanning resolution of 20 µm, indicating the effectiveness of the applied HIP process.

The analysis of the tomographic images revealed the presence of microcracks, whose propagation correlated with the printing orientation and the build direction. In samples printed in the XY orientation, the cracks propagated along the longitudinal direction of the specimens (Figure 16), whereas in samples printed in the XZ orientation, crack propagation occurred in the transverse direction (Figure 17). This behavior is consistent with mechanisms reported in the literature [36,37], which indicate that cracks tend to propagate along specific paths, often aligning with the raster directions or print layers. This behavior is influenced by the anisotropic nature of LPBF-printed samples and mechanical properties of the material.

### 3.3. Chemical Composition

**Figure 18:**
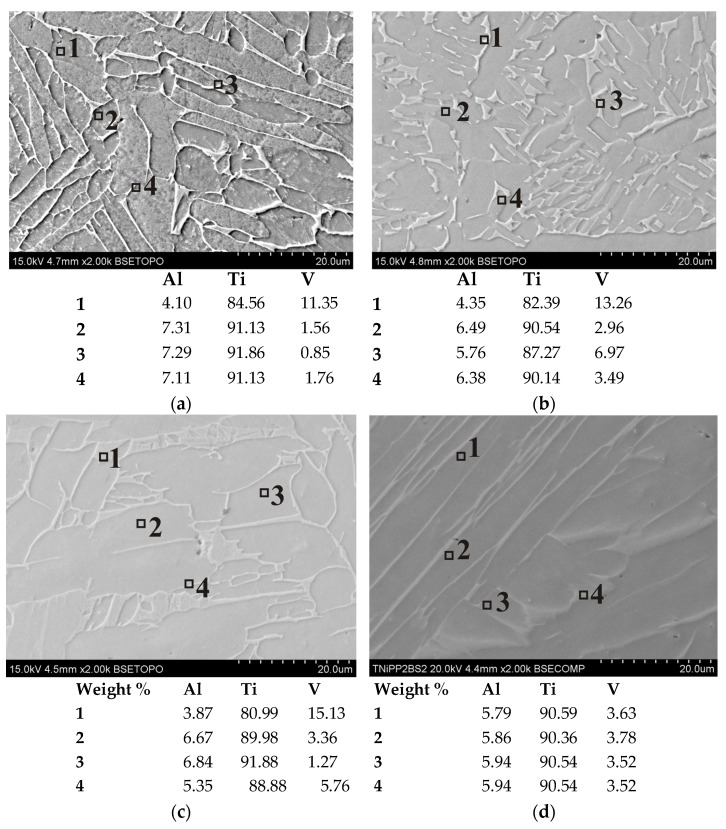

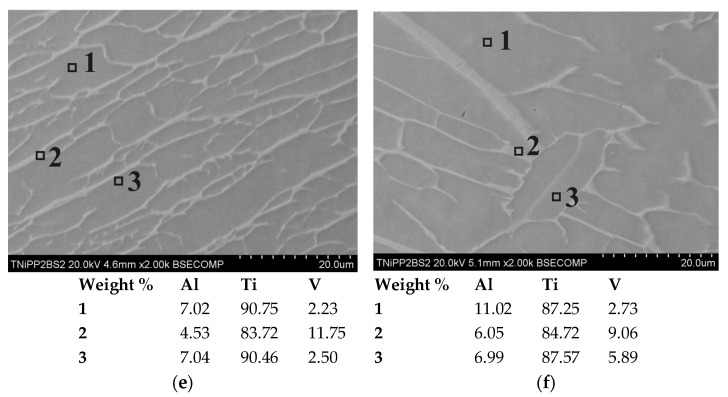
Spot EDS analysis results for samples (**a**) 1XY, (**b**) 2XY, (**c**) 4XY-HT, (**d**) 5XZ, (**e**) 7XZ-HT, and (**f**) 8XZ-HT.

Spot EDS analysis performed on selected samples (Figure 18) allowed for the assessment of the homogeneity of the alloying element distribution. In all cases, titanium and aluminum are the dominant components, forming the metallic matrix. Vanadium, on the other hand, tends to localize at the grain boundaries. In the as-printed 1XY sample (Figure 18a), the vanadium content ranged from 0.85 to 11.35 wt.%, indicating the presence of local microsegregation in the distribution of Ti, Al, and V. In the 2XY sample (Figure 18b) subjected to HIP and TCAP, the vanadium content ranged between 2.96 and 13.26 wt.%, suggesting that even after severe plastic deformation, segregation was not fully reduced. The 4XY-HT sample subjected to HIP at 1250 °C and heat treatment was also characterized by an unstable vanadium distribution (1.27–15.13 wt.%) with a more uniform Ti and Al content (Figure 18c). In the 5XZ sample (Figure 18d), the vanadium content was very stable and remained in the range of 3.63–3.78 wt.%. In the 7XZ-HT (Figure 18e) and 8XZ-HT (Figure 18f) samples subjected to HIP and heat treatment, clear differences in vanadium concentration were observed: from 2.23 to 11.75 wt.% (7XZ-HT) and from 2.73 to 9.06 wt.% (8XZ-HT). This indicates the phenomenon of microsegregation of elements during crystallization and varying degrees of homogenization depending on the HIP parameters [38].

### 3.4. Microhardness

**Figure 19:**
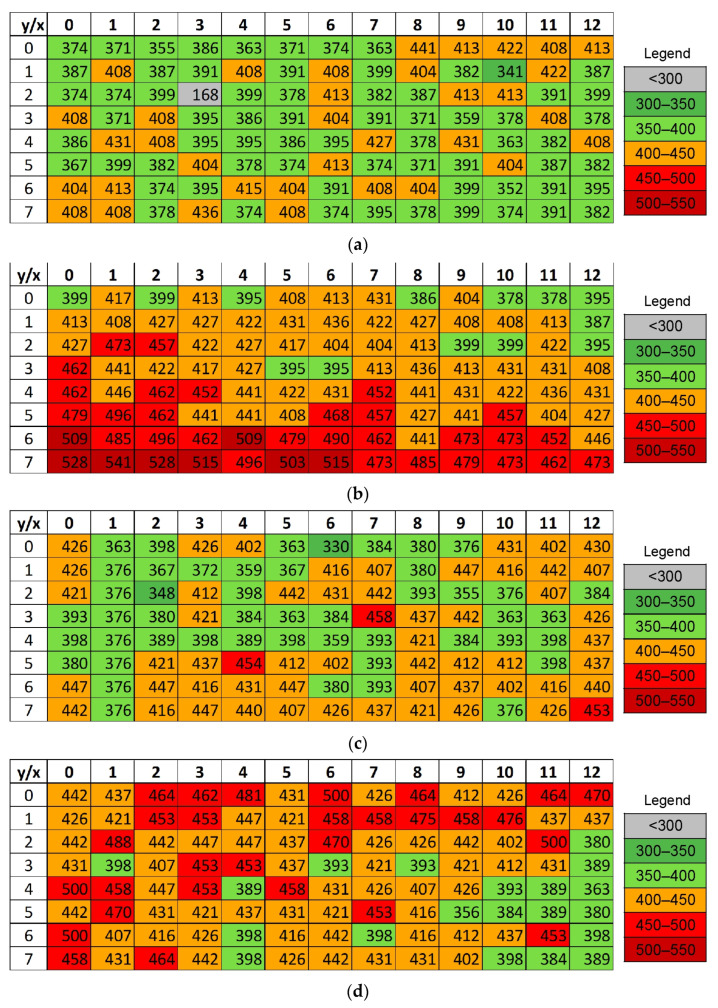

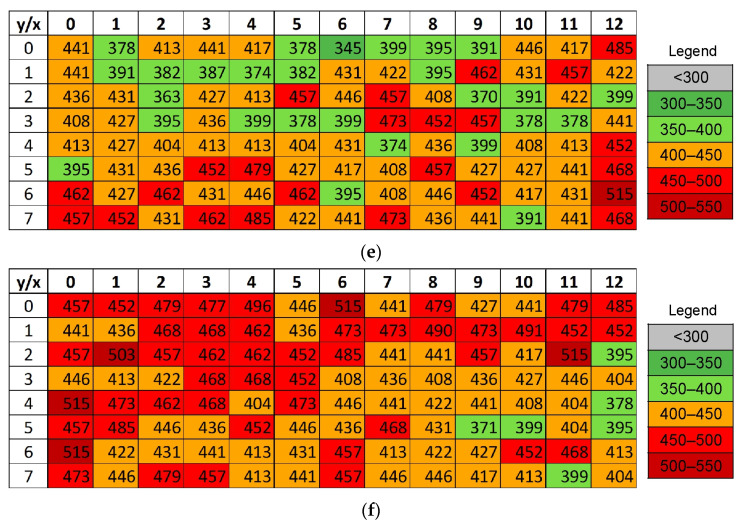
Microhardness (HV0.5) measurements in a rectangular grid mid-way along the transverse section of the following samples: (**a**) 1XY, (**b**) 2XY, (**c**) 3XY, (**d**) 4XY, (**e**) 3XY-HT, and (**f**) 4XY-HT.

**Figure 20:**
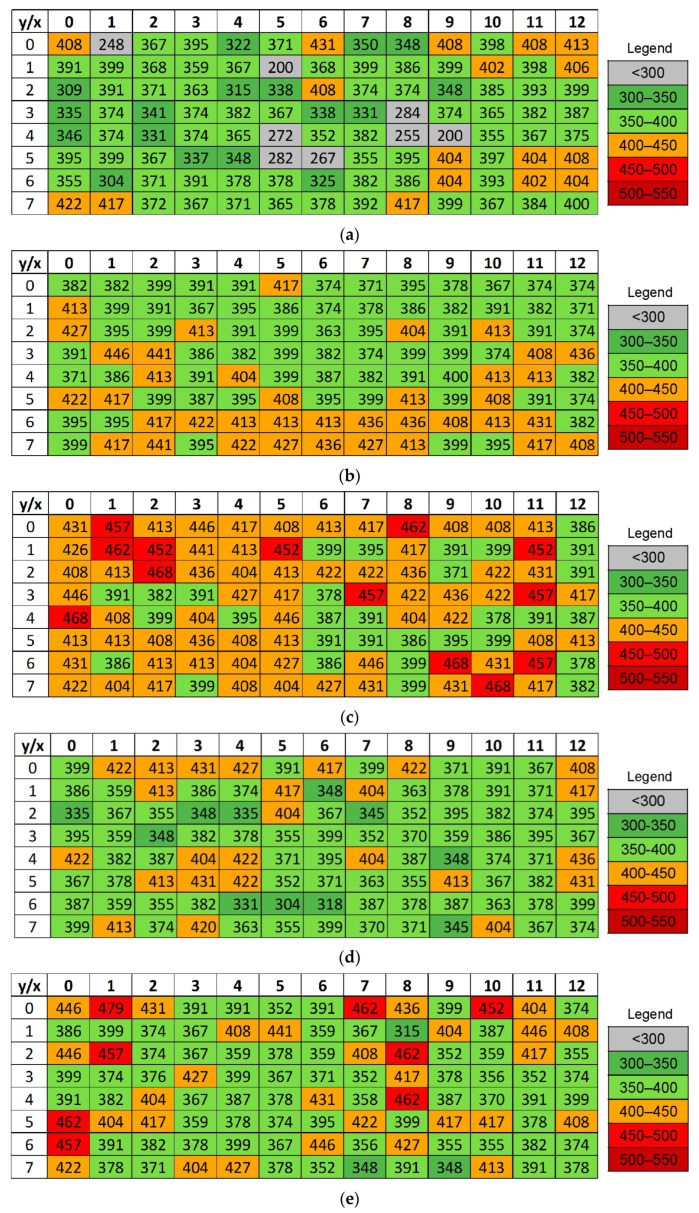

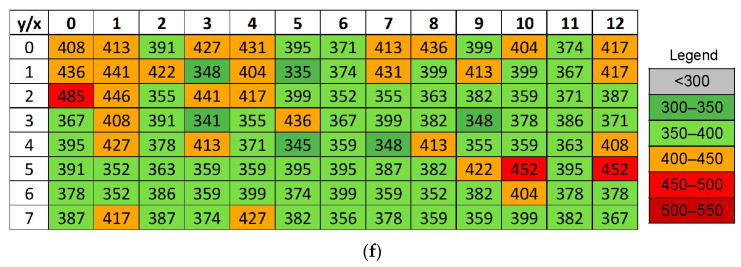
Microhardness (HV0.5) measurements in a rectangular grid mid-way along the cross-section of the following samples: (**a**) 5XZ, (**b**) 6XZ, (**c**) 7XZ, (**d**) 8XZ, (**e**) 7XZ-HT, and (**f**) 8XZ-HT.

Microhardness measurements were performed in a rectangular grid x × y (where x is the width and y is the height of the sample section) in the middle of the transverse section of the samples. To observe changes in the work hardening of the samples, microhardness maps were generated separately for the samples printed in the XY plane and subjected to HIP at 1150 °C and 1250 °C. Such maps were also generated for the corresponding samples printed in the XZ plane after HIP, and after HIP and heat treatment. The microhardness results for samples printed in the XY and XZ planes are presented in Figure 19 and Figure 20.

Microhardness distribution analysis shows a clear correlation between the temperature of the hot isostatic pressing process and microhardness. In the samples printed in the XY-plane, the LPBF layers are aligned parallel to the load direction during microhardness measurement, which promotes a more uniform stress distribution and results in a more uniform microhardness distribution compared to samples printed in the XZ plane. As the HIP temperature increases, a systematic increase in microhardness was observed, which is associated with the decomposition of the α’ martensite and the formation of a stable α + β microstructure [39]. The microhardness of the 1XY sample in the as-printed condition does not exceed 450 HV0.5 (Figure 19a). The TCAP process preceded by HIP treatment resulted in a significant increase in microhardness, as expected (Figure 19b). The microhardness was only lower than 400 HV0.5 at a few points. The HIP process further reduces porosity and removes microcracks formed during the LPBF process, leading to improved material homogeneity and increased hardness [40].

Further strengthening of the samples occurred as a result of additional heat treatment after HIP. As the HIP temperature increased, the microhardness of samples subjected to the subsequent heat treatment also increased (Figure 19d,f). Annealing under appropriately selected conditions favors the precipitation of fine particles of the α phase in the β phase, leading to a strengthening effect by precipitation, which was also observed in [41]. Consequently, samples printed in the XY plane, which were subjected to HIP and additional annealing, achieved higher microhardness values than samples which were only subjected to HIP.

The microhardness measurement obtained for the sample subjected to the TCAP process, carried out after annealing at 910 °C, is of particular note. The microhardness map (Figure 19b) shows increasing microhardness values resulting from the work hardening phenomenon. These microhardness values exceed the maximum values of microhardness obtained for samples subjected to HIP (Figure 19c,d) or HIP with additional heat treatment (Figure 19e,f). However, such a large increase in microhardness had a negative effect and the material lost its plastic properties, which led to the sample cracking during the TCAP process. Excessive hardening of Ti6Al4V alloys in SPD processes can lead to a rapid reduction in plasticity and an increase in crack initiation [42].

The microhardness distribution maps of the samples printed in the XZ plane confirm that samples in the as-printed state were characterized by high heterogeneity (Figure 20) and the presence of α’ martensite. Analyses of maps reveals characteristic differences compared to samples printed in the XY plane (Figure 19). In the XZ plane, the 3D printing direction is perpendicular to the analyzed cross-section, which favors the formation of distinct thermal gradients along the printing direction. This results in greater inhomogeneity in the microhardness distribution and the presence of a banded structure resulting from the deposition of successive layers. Similarly to 1XY sample (Figure 19a), the microhardness maps show areas with microhardness below 300 HV0.5 (Figure 20a), which correspond to the presence of pores in the microstructure. Moreover, the number of areas where they occurred was higher, which was related not only to pores but also to the presence of holes, probably resulting from the appearance of microcracks during the TCAP process. The HIP process leads to a more pronounced homogenization of microstructure and defect reduction, with the morphology of the microstructure and microhardness distribution being dependent on the HIP process temperature. This effect was also observed in [20,43]. HIP at 910 °C significantly reduced defects and porosity, resulting in a more uniform microhardness distribution and the absence of pore-related values below 300 HV0.5 (Figure 20b). Additional heat treatment affects the microstructure in various ways. In samples which were HIP-processed at 1150 °C, annealing promotes further decomposition of the α’ phase and a decrease in microhardness, whereas in samples HIP-processed at 1250 °C, it can lead to secondary hardening through the precipitation of the α phase in the β phase matrix. These observations indicate that larger thermal gradients occurred in the samples printed in the XZ plane, and the presence of martensite results in stronger heterogeneity in the material properties. The combined HIP and annealing processes play a key role in their stabilization [44].

**Figure 21:**
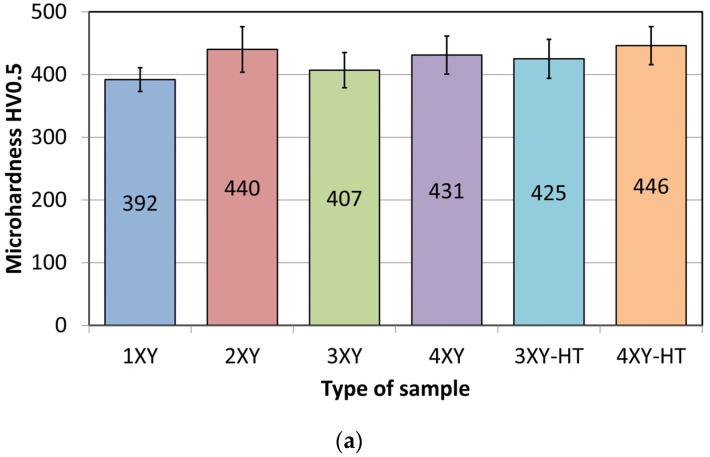

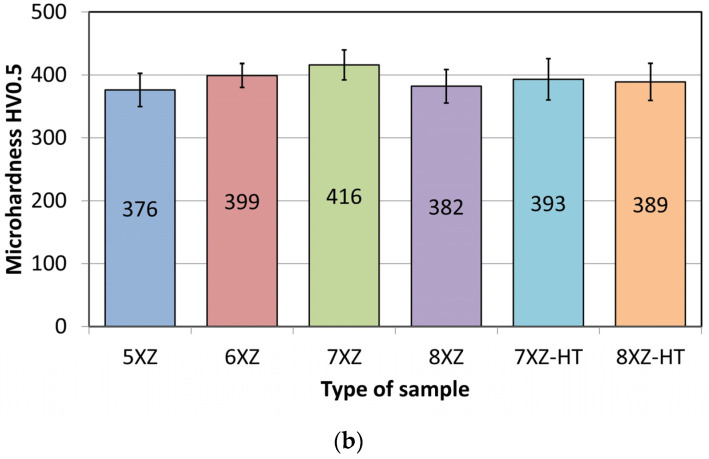
Vickers microhardness for Ti6Al4V samples printed in (**a**) XY and (**b**) XZ plane.

Figure 21 shows the average hardness values of the tested samples. The results clearly indicate that both HIP temperature and the use of additional treatment (annealing, TCAP) have a significant impact on the microhardness values, and the microhardness range, in LPBF-processed Ti6Al4V samples. In the XY printing plane, the best results were obtained for the sample after HIP at 1250 °C and heat treatment, where the average microhardness reached the highest value (Figure 21a). However, the increase in the average values of 3XY-HT and 4XY-HT hardness was also associated with a less uniform microhardness distribution, particularly across the sample height (Figure 19e,f). In the XZ printing plane, the HIP process performed at 910 °C proved to be particularly beneficial, providing the smallest distribution of hardness values, despite its lower average value (Figure 21b). The comparison of both printing orientations demonstrates that printing orientation significantly determines the effectiveness of HIP and heat treatment processes, and their optimization must be tailored to the component’s manufacturing direction.

Additional compression test results for samples after printing, without additional postprocessing, have been added in Table 4. Samples with a square cross-section of 5 × 5 mm and a height of 6.5 mm were compressed at a constant strain rate of 3 × 10^−3^ s^−1^. The test was carried out on XZ-type samples, which were compressed in the longitudinal and transverse directions to the 3D printing direction. In the case of the analyzed material, slight differences in the values of mechanical properties were observed, with higher yield strength and compressive strain obtained for the transverse direction.

## 4. Conclusions

The results presented in this paper allowed for a multifaceted analysis of the microstructure, porosity, and microhardness of Ti6Al4V samples fabricated using the LPBF process, which were then subjected to the HIP process, heat treatment, and TCAP tests. Based on the experimental studies, the following conclusions can be drawn:

- Three-dimensional printing direction was found to have a significant effect on the microstructure and microhardness of the samples. Samples printed in the XY plane were characterized by a more uniform arrangement of overlapping layers, a uniform microstructure, and lower porosity. However, samples printed in the XZ plane exhibited distinct interlayer boundaries and local discontinuities, resulting in greater microstructural anisotropy and a higher risk of microcracks. The microhardness distribution maps of the samples printed in the XZ plane confirm that samples in the as-printed state were characterized by high heterogeneity and the presence of α’ martensite.
- The HIP process played a key role in reducing porosity and homogenizing the microstructure. Due to isostatic pressure and high temperatures, diffusion sealing of the internal porosity and reduced discontinuities were achieved. As a result, porosity significantly decreased, and the microstructure became more uniform. This translated into an increase in the microhardness of the LPBF-processed Ti6Al4V material, although a small number of defects still remained.
- Heat treatment after HIP proved equally important in further improving the properties of 3D-printed samples. Annealing favors the precipitation of fine particles of the α phase in the β phase, leading to a strengthening effect by precipitation. In samples printed in the XY plane, additional annealing led to the disintegration of the metastable α’ phase and the formation of the more stable α + β phases. The samples subjected to HIP and heat treatment were characterized by a more homogeneous structure and finer grains than the as-printed samples. In samples printed in the XZ plane, the effects of annealing were less clear; in some sample variants, the microstructure and porosity improved but local inhomogeneity in the microhardness measurement results was observed.
- The annealing operation performed after HIP was insufficient to lower the material hardness of the samples before the TCAP process. The tested samples were fractured as a result of the severe plastic deformation. Potential causes of excessive work hardening include the microsegregation of vanadium at grain boundaries, rapid cooling leading to α’ crystallization, high thermal gradients in the XZ print orientation, and the effect of overlapping scan tracks, which generated inhomogeneous stress fields. Future research is planned to explore the back pressure equal channel angular pressing method, which, by introducing back pressure, could improve the formability of the workpiece by reducing stress concentration and the risk of cracking. Further research should also focus on further optimization of the LPBF process parameters (higher laser power, different scanning strategies, etc.), adjusting the temperature and conditions of the SPD process, as well as hybrid postprocessing sequences that effectively control the microstructure and properties of the LPBF-fabricated Ti6Al4V titanium alloy samples.

## Figures and Tables

**Table 1:** Technological parameters of the SLM process.

Parameter	Value
Laser power	400 W
Scanning speed	800 mm/s
Laser spot diameter	0.075 mm
Scanning strategy	Strip hatching
Hatching distance	0.1 mm
Layer thickness	0.06 mm
Preheating temperature	170 °C

**Table 2:** Research plan.

3D Printing Orientation	Sample Designation	Postprocessing Method of As-Printed Samples			
		No Post-Printing Methods	HIP	HT	TCAP
XY	1XY	✓			
	2XY		✓ (910 °C)		✓
	3XY-HT		✓ (1150 °C)	✓	
	3XY		✓ (1150 °C)		
	4XY-HT		✓ (1250 °C)	✓	
	4XY		✓ (1250 °C)		
XZ	5XZ				✓
	6XZ		✓ (910 °C)		
	7XZ-HT		✓ (1150 °C)	✓	
	7XZ		✓ (1150 °C)		
	8XZ-HT		✓ (1250 °C)	✓	
	8XZ		✓ (1250 °C)		

**Table 3:** Chemical composition of the Ti6Al4V powder (wt.%).

Al	V	O	C	N	H	Fe	Ti
5.5–6.5	3.5–4.5	<0.2	<0.08	<0.05	<0.015	<0.25	balance

**Table 4:** Basic mechanical parameters of 3D-printed samples obtained from a compression test.

Parameter	Sample Orientation Relative to the 3D Printing Direction	
	Longitudinal	Transverse
Yield strength R_c0.2_, MPa	1019	1061
Compressive strength R_m_, MPa	1688	1679
Compressive strain A_c_, %	2.4	3.3

## Data Availability

The original contributions presented in this study are included in the article. Further inquiries can be directed to the corresponding author.

## References

[1] Fan Z., Feng H. (2018). Study on selective laser melting and heat treatment of Ti-6Al-4V alloy. Results Phys..

[2] Lütjering G. (1998). Influence of processing on microstructure and mechanical properties of (α + β) titanium alloys. Mater. Sci. Eng. A.

[3] Liu S., Shin Y.C. (2019). Additive manufacturing of Ti6Al4V alloy: A review. Mater. Des..

[4] Nguyen H.D., Pramanik A., Basak A.K., Dong Y., Prakash C., Debnath S., Shankar S., Jawahir I.S., Dixit S., Buddhi D. (2022). A critical review on additive manufacturing of Ti-6Al-4V alloy: Microstructure and mechanical properties. J. Mater. Res. Technol..

[5] Yang J., Yu H., Yin J., Gao M., Wang Z., Zeng X. (2016). Formation and control of martensite in Ti-6Al-4V alloy produced by selective laser melting. Mater. Des..

[6] Żaba K., Madej M., Leszczyńska-Madej B., Trzepieciński T., Sitek R. (2025). Tribological Performance of Direct Metal Laser Sintered 20MnCr5 Tool Steel Countersamples Designed for Sheet Metal Forming Applications. Appl. Sci..

[7] Al-Bermani S., Blackmore M., Zhang W., Todd I. (2010). The origin of microstructural diversity, texture, and mechanical properties in electron beam melted Ti-6Al-4V. Metall. Mater. Trans. A.

[8] Hu Y., Chen H., Jia X., Liang X., Lei J. (2022). Heat treatment of titanium manufactured by selective laser melting: Microstructure and tensile properties. J. Mater. Res. Technol..

[9] Żaba K., Balcerzak M., Kuczek Ł., Wiewióra M., Różycka I., Trzepieciński T., Mizera J. (2024). Application of Powder-Bed Fusion of Metals Using a Laser for Manufacturing of M300 Maraging Steel Tools Intended for Sheet Metal Bending. Materials.

[10] Żaba K., Szczepańska M., Balcerzak M., Kac S., Żabinski P. (2025). Assessment of the Corrosion Rate of Maraging Steel M350 Produced by Additive Manufacturing Using the Laser Powder-Bed Fusion Method and Surface Finishing Techniques. Materials.

[11] Sun W., Ma Y., Huang W., Zhang W., Qian X. (2020). Effects of build direction on tensile and fatigue performance of selective laser melting Ti6Al4V titanium alloy. Int. J. Fatigue.

[12] Hao Y.-L., Li S.-J., Yang R. (2016). Biomedical titanium alloys and their additive manufacturing. Rare Met..

[13] Carroll B.E., Palmer T.A., Beese A.M. (2015). Anisotropic tensile behavior of Ti-6Al-4V components fabricated with directed energy deposition additive manufacturing. Acta Mater..

[14] Hrabe N., Gnäupel-Herold T., Quinn T. (2017). Fatigue properties of a titanium alloy (Ti–6Al–4V) fabricated via electron beam melting (EBM): Effects of internal defects and residual stress. Int. J. Fatigue.

[15] Ricci S., Iannitti G. (2024). Mechanical Behavior of Additive Manufacturing (AM) and Wrought Ti6Al4V with a Martensitic Microstructure. Metals.

[16] Voisin T., Calta N.P., Khairallah S.A., Forien J.-B., Balogh L., Cunningham R.W., Rollett A.D., Wang M. (2018). Defects-dictated tensile properties of selective laser melted Ti-6al-4V. Mater. Des..

[17] Sabban R., Bahl S., Chatterjee K., Suwas S. (2019). Globularization using heat treatment in additively manufactured Ti-6Al-4V for high strength and toughness. Acta Mater..

[18] Swarnakar A.K., Van Der Biest O., Baufeld B. (2011). Thermal expansion and lattice parameters of shaped metal deposited Ti-6Al-4V. J. Alloys Compd..

[19] Cordero Z.C., Knight B.E., Schuh C.A. (2016). Six decades of the Hall-Petch effect—A survey of grain-size strengthening studies on pure metals. Int. Mater. Rev..

[20] Lee D., So T.Y., Yu H.A., Kim G., Moon E., Ko S.H. (2024). Effect of hot isostatic pressing and solution heat treatment on the microstructure and mechanical properties of Ti-6Al-4V alloy manufactured by selective laser melting. Arch. Metall. Mater..

[21] Beese A.M., Carroll B.E. (2016). Review of Mechanical Properties of Ti-6Al-4V Made by Laser-Based Additive Manufacturing Using Powder Feedstock. JOM.

[22] Liang Z., Sun Z., Zhang W., Wu S., Chang H. (2019). The effect of heat treatment on microstructure evolution and tensile properties of selective laser melted Ti6Al4V alloy. J. Alloys Compd..

[23] Tanski T., Snopinski P., Pakiela W., Borek W., Prusik K., Rusz S. (2016). Structure and Properties of AlMg Alloy after Combination of ECAP and Post-ECAP Ageing. Arch. Civ. Mech. Eng..

[24] Dutkiewicz J., Rusz S., Kuc D., Hilser O., Pałka P., Boczkal G. (2017). Superplastic deformation of two phase MgLiAl alloy after TCAP pressing. Acta Metall. Slovaca.

[25] Koujalagi M.B., Siddesha H.S. (2021). ECAP of titanium alloy by sever plastic deformation: A review. Mater. Today Proc..

[26] Rusz S., Hilser O., Ochodek V., Cada R., Svec J., Szkandera P. (2019). Influence of SPD Process on Low-Carbon Steel Mechanical Properties. MM Sci. J..

[27] Bartolomeu F., Faria S., Carvalho O., Pinto E., Alves N., Silva F., Miranda G. (2016). Predictive models for physical and mechanical properties of Ti6Al4V produced by Selective Laser Melting. Mater. Sci. Eng. A.

[28] Rusz S., Cizek L., Salajka M., Tylsar S. (2014). Ultrafine grain refinement of AlMn1Cu and AZ 31 alloys by spd process. Arch. Metall. Mater..

[29] (2018). Metallic Materials—Vickers Hardness Test Part 1—Test Method.

[30] Mayer T., Friso F., Radis R. (2025). Effect of Build Orientation on Thermal Expansion of LPBF Printed Ti-6Al-4V. Metall. Mater. Trans..

[31] Wu M.W., Ni K., Yen H.W., Chen J.K., Wang P., Tseng Y.-J., Tsai M.-K., Wang S.-H., Lai P.-H., Ku M.-H. (2023). Revealing the intensified preferred orientation and factors dominating the anisotropic mechanical properties of laser powder bed fusion Ti-6Al-4V alloy after heat treatment. J. Alloys Compd..

[32] Mendoza I., Villalobos D., Alexandrov B.T. (2015). Crack propagation of Ti alloy via adiabatic shear bands. Mater. Sci. Eng. A.

[33] Liu Y., Chen F., Xu G., Cui Y., Chang H. (2020). Correlation between Microstructure and Mechanical Properties of Heat-Treated Ti–6Al–4V with Fe Alloying. Metals.

[34] Karasoglu M., Öteyaka M.Ö., Yasa E., Tan E., Kuşhan M.C. (2024). Effect of Heat Treatment and Hot Isostatic Pressing on the Corrosion Behavior of Ti6Al4 V Parts Produced by Electron Beam Melting Additive Manufacturing Technology. ACS Omega.

[35] Ojo S.A., Bowser B., Manigandan K., Morscher G.N., Dong Y., Gyekenyesi A.L., Scott-Emuakpor O.E. (2023). Improving fatigue life of additively repaired Ti-6Al-4V subjected to laser-assisted ultrasonic nanocrystal surface modification. Int. J. Fatigue.

[36] Bragaglia M., Cecchini F., Paleari L., Ferrara M., Rinaldi M., Nanni F. (2023). Modeling the fracture behavior of 3D-printed PLA as a laminate composite: Influence of printing parameters on failure and mechanical properties. Compos. Struct..

[37] Irani M.S., Ranjbar S., Lakhi M., Zolfagharian A. (2025). Enhancing damage tolerance of structures using 3D/4D printing technologies. Adv. Mater. Technol..

[38] Zheng H., Bai Y., Hou W., Zha Z., Zhang L.C., Li S., Yang R. (2025). The significant impact of hot isostatic pressing on the corrosion behavior of Ti-6Al-4V alloy prepared by electron beam powder bed fusion. npj Mater. Degrad..

[39] Xu W., Brandt M., Sun S., Elambasseril J., Liu Q., Latnam K., Xia K., Qian M. (2015). Additive manufacturing of strong and ductile Ti–6Al–4V by selective laser melting via in situ martensite decomposition. Acta Mater..

[40] Squillaci L., Neikter M., Hansson T., Pederson R., Moverare J. (2025). Microstructure and mechanical properties of Ti-6Al-4V alloy fabricated using powder bed fusion—Laser beam additive manufacturing process: Effect of hot isostatic pressing. Mater. Sci. Eng. A.

[41] Li T., Kent D., Sha G., Dargusch M.S., Cairney J.M. (2014). Precipitation of the α-phase in an ultrafine grained beta-titanium alloy processed by severe plastic deformation. Mater. Sci. Eng. A.

[42] Karaman I., Yapici G.G. Effect of Severe Plastic Deformation on the Mechanical Behavior of Ti-6Al-4V. Proceedings of the ASME 2003 International Mechanical Engineering Congress and Exposition.

[43] Sheng K., Liu W., Wang N., Li B., Chen L., Liu B., Ren D., Zhu J., Yin M. (2025). Microstructure and mechanical properties of Ti–6Al–4V fabricated by electron beam powder bed fusion regulated via hot isostatic pressing. J. Mater. Res. Technol..

[44] Sha W., Malinov S. (2009). Titanium Alloys: Modelling of Microstructure, Properties and Applications.

